# Port site tuberculosis after laparoscopic cholecystectomy in resource limiting setup: Case series and literature review

**DOI:** 10.1016/j.ijscr.2024.110722

**Published:** 2024-12-08

**Authors:** Mulugeta Wondmu Kedimu, Addisu Assfaw Ayen, Zemen Asmare Emiru, Yoseph Gebremedhin Kassie, Aklog Almaw Yigzaw, Amsalu Molla Getahun

**Affiliations:** a Department of surgery, Debre Tabor University, Debre Tabor, Ethiopia; b Department of Internal Medicine, Debre Tabor University, Debre Tabor, Ethiopia; c Department of Pathology, Debre Tabor University, Debre Tabor, Ethiopia; d Department of internal medicine, Debre Tabor comprehensive specialized hospital, Debre Tabor, Ethiopia

**Keywords:** Laparoscopic cholecystectomy, Port site infection, Port site tuberculosis (TB), Extrapulmonary TB, Case series, Ethiopia

## Abstract

**Introduction and importance:**

Laparoscopic cholecystectomy has become the gold standard for treating symptomatic gallstones in recent years due to its minimally invasive nature, which results in less pain, scarring, and a faster recovery time compared to traditional open surgery. Port site infection is a rare complication of laparoscopic surgery, sometimes occurring early after the procedure or developing later and the cause of these infections can vary. Port site tuberculosis (TB) is a particularly uncommon type of port site infection and represents a rare form of extra pulmonary TB.

**Case presentation:**

Three patients (a 63-year-old diabetic male, a 62-year-old hypertensive female, and a 34-year-old female) presented with port site discharge and pain after undergoing laparoscopic cholecystectomy at the same center during the same campaign. All patients initially received antibiotics for suspected port site infections, but these failed to resolve the symptoms. Subsequent fine needle aspiration cytology revealed port site tuberculosis (TB) in all three cases. After initiating anti-tuberculosis treatment, all patients showed significant improvement.

**Clinical discussion:**

Port site tuberculosis, a rare and often overlooked extra pulmonary manifestation of tuberculosis, can present as a port site infection following laparoscopic procedures. It is characterized by prolonged wound healing and persistent discharge, can pose diagnostic challenges, often requiring careful evaluation and appropriate investigations to ensure timely and effective treatment. Port site tuberculosis can be transmitted to patients through two main routes: exogenous or endogenous ways. Once a diagnosis of port site tuberculosis is confirmed; the next step involves treatment with a standard anti-tuberculosis regimen.

**Conclusion:**

Port site tuberculosis (TB) after undergoing laparoscopic cholecystectomy at the same facility, suggesting inadequate instrument sterilization as a likely cause. This case underscores the importance of considering port site TB in post-laparoscopic patients, particularly in areas with high TB prevalence, after ruling out bacterial infections. Prompt and appropriate treatment is crucial for successful outcomes.

## Highlights

• Rare surgical site infection
• Rare extra pulmonary type tuberculosis
• Diagnosis challenge
• Laparoscopic instrument sterilization is required for all patients who undergone laparascopic procedure.

## 1 Introduction

Laparoscopic surgery, initially developed in the 1960s primarily for diagnostic purposes, transitioned to become a valuable tool for both therapeutic and diagnostic procedures in the 1980s, revolutionizing surgical practices and improving patient outcomes (1). Laparoscopic cholecystectomy has become the gold standard for treating symptomatic gallstones in recent years due to its minimally invasive nature, which results in less pain, scarring, and a faster recovery time compared to traditional open surgery (2). Despite the inherent risk of infection associated with any surgical wound, laparoscopic procedures have a lower infection rate compared to open surgical treatments (3,4). Port site infection is a rare complication of laparoscopic surgery, sometimes occurring early after the procedure or developing later and the cause of these infections can vary (5). Port site tuberculosis (TB) is a particularly uncommon type of port site infection and represents a rare form of extra pulmonary TB (6). This report describes three patients a 63-year-old male, a 52-year-old female, and a 27-year-old female; who all developed port site TB after undergoing laparoscopic cholecystectomy at the same medical center; retrospective case series.

This case series narrated with PROCESS criteria (7).

## 2 Case presentations

### 2.1 Case one

A 63-year-old male patient from Debre Tabor, Ethiopia, presented to the outpatient department. He is a known type II diabetes, for which he takes metformin 1 g orally twice daily, and hypertension, for which he takes amlodipine 10 mg orally daily. His presenting complaint was discharge from the surgical port sites, which had been present for one month following a laparoscopic cholecystectomy. The discharge was whitish and thick on all four port sites, with adjacent skin color change. Otherwise, he had no fever, cough, or pain. On examination, vital signs were normal, and abdominal examination showed a cheese-like discharge from four port sites. The other examination was normal.

On investigations; the complete blood count (CBC) showed a white blood cell count of 7700/L, with 35.2 % granulocytes and 59.5 % lymphocytes. Hemoglobin was 14.5 g/dl, and platelets were 329,000 cells. The erythrocyte sedimentation rate (ESR) was 65 mm/h. All organ function tests and serum electrolytes were within the normal range. The hemoglobin A1c level was 7.5 %. HIV serostatus, Gram stain, and acid-fast bacilli were all negative. An abdominal ultrasound revealed multiple port site collections, the largest measuring 2.6 cm in depth.

A diagnosis of surgical site infection was initially suspected, and the patient received wound debridement and daily wound care. Augmentin was prescribed for one week, and wound care was continued. Despite the patient taking the antibiotics as instructed, there was no improvement, and he continued to experience wound discharge and pain.

**Fig. 1:**
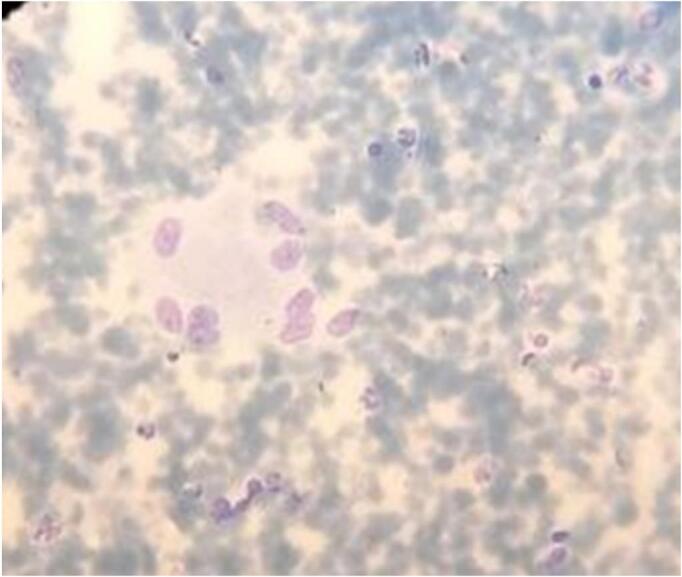
Epitheliod cell aggregates on hemorrhagic background.

At a follow-up one month later, the patient's symptoms had not improved. Repeat discharge analysis revealed negative Gram stain and AFB stains. However, a fine needle aspiration cytology (FNAC) of the abdominal wall showed granulomatous inflammation, as depicted in Fig. 1.

Then a diagnosis of port site tuberculosis was made, and the patient was started on anti-tuberculosis therapy with RHZE for two months, followed by RH for four months. His existing medications, metformin and amlodipine, were continued throughout treatment. Subsequently, the discharge resolved, and the wound healed completely. Currently, the patient has no discharge, and the wound is well-scarred.

### 2.2 Case two

A 62-year-old female patient with a history of hypertension, managed with lifestyle modifications, presented to the clinic with a three-month history of discharge from her surgical port sites following a laparoscopic procedure. The discharge was whitish and present at all port sites. The laparoscopic cholecystectomy was performed for symptomatic cholelithiasis. The patient denied experiencing cough, chest pain, weight loss, night sweats, or fever. She reported having visited multiple hospitals for this complaint and had been prescribed various medications, but she could not recall the specific names with daily wound care.

**Fig. 2:**
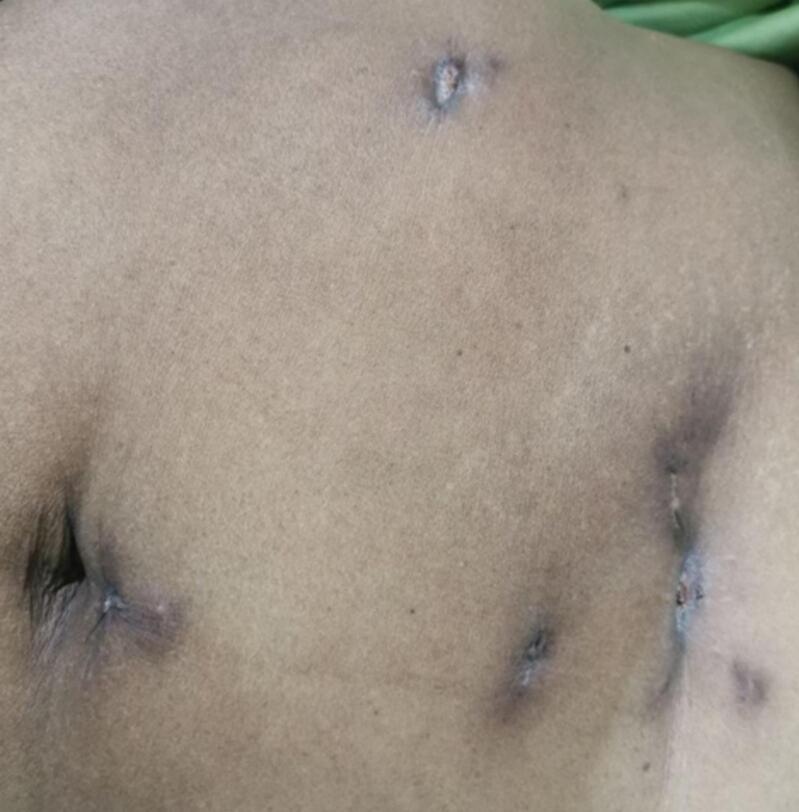
Nodular swelling over laparoscopic sites.

The patient appeared acutely ill with mild discomfort. Her vital signs were all within the normal range, except for her axillary temperature, which was 39.9 °C. Abdominal examination revealed multiple sinus tracts with whitish discharge at the previous port sites. The edges of the wounds were firm, raised, and measured approximately 1 cm in diameter, as shown in Fig. 2. The remainder of the physical examination was unremarkable.

On Investigations: the patient's complete blood count (CBC) showed a white blood cell count of 5200/μL, with 54.6 % granulocytes and 39.6 % lymphocytes. Her hemoglobin level was 14.8 g/dL, and her platelet count was 213,000/μL. The erythrocyte sedimentation rate (ESR) was 39 mm/h. All organ function tests and serum electrolytes were within the normal range. Further investigations revealed that the patient's HIV serostatus was negative. An abdominal ultrasound showed multiple port site collections, the largest measuring 2.3 cm × 1.8 cm in depth.

Then the patient was diagnosed with port site infection. Incision and drainage were performed under local anaesthesia at all three port sites. Frank pus was drained and sent for culture and sensitivity testing, which showed no growth. Gram stain, acid-fast bacilli (AFB and culture of the drainage were also negative. Given the similarity of this patient's presentation to Case 1, who had undergone a laparoscopic procedure in the same time frame, a fine needle aspiration cytology (FNAC) of the abdominal wall was performed. This revealed granulomatous inflammation with central necrosis.

Following that, anti-tuberculosis therapy (2RHZE/4H regimen) with pyridoxine was initiated, based on the diagnosis of port site tuberculosis. Wound care was continued as well. Within one month, the wound discharge had significantly improved. With continued anti-tuberculosis therapy, the wound healed completely upon completion of the treatment regimen.

### 2.3 Case three

A 34-year-old female patient from Debre Markos underwent a standard four-port laparoscopic cholecystectomy for cholelithiasis at the same center as the previous case during the same campaign. The surgery was uneventful. The patient was discharged home postoperatively. Ten days later, she presented with minimal discharge from the umbilical wound. She was advised to have daily wound care and prescribed Augmentin for five days at a local private clinic. Three weeks later, the patient returned, reporting increasing wound discharge and pain at all port sites. She denied experiencing fever, cough, or weight loss.

The patient was conscious and not in respiratory distress. Her vital signs were stable, with a blood pressure of 110/70 mmHg, a pulse rate of 90 beats per minute, a temperature of 36.9 °C, and a respiratory rate of 20 breaths per minute. The rest of her physical examination was unremarkable except for her abdomen. Abdominal examination revealed tenderness and swelling at the port sites, along with wound discharge.

The patient's complete blood count (CBC) showed a white blood cell count of 6400/μL, with 64.6 % granulocytes and 28.9 % lymphocytes. Her hemoglobin level was 13.8 g/dL, and her platelet count was 295,000/μL. The erythrocyte sedimentation rate (ESR) was 45 mm/h. All organ function tests and serum electrolytes were within the normal range. Further investigations revealed that the patient's HIV serostatus was negative. The Gram stain and culture of the wound discharge were negative, but the acid-fast bacilli (AFB) stain was positive for acid-fast bacilli.

The patient was diagnosed with port site TB at Debre Markos Specialized Hospital and received treatment with a standard 2RHZE/4RH regimen, along with wound care and nutritional rehabilitation. She adhered to her medication, and at the one-month follow-up, while still experiencing pain, her wound discharge had improved. By the two-month follow-up, all symptoms had improved, and the wound had healed completely. Having successfully completed her anti-TB treatment, the patient is now well and fully able to participate in her daily activities.

## 3 Discussion

Port site tuberculosis, a rare and often overlooked extra pulmonary manifestation of tuberculosis, can present as a port site infection following laparoscopic procedures (8). It is characterized by prolonged wound healing and persistent discharge, can pose diagnostic challenges, often requiring careful evaluation and appropriate investigations to ensure timely and effective treatment (9).

Surgical site infections (SSIs), occurring after any surgical procedure, are among the most common hospital-acquired infections and often complicate patient recovery (10). Surgical site infections after laparoscopic surgery (port site infection) are most common early in the postoperative period, typically caused by gram-positive or gram-negative bacteria. However, late-onset infections can also develop, often stemming from chronic infections like mycobacterial or non-mycobacterial sources (6). Due to its minimally invasive nature, laparoscopic surgery typically has a lower rate of surgical site infections compared to open surgical procedures (11). Even though the rate of port site infection can vary, a study by Sadia S. Siddiqua et al. found a rate of 3.92 % among 1478 laparoscopic cholecystectomy patients (8).

Several factors have been linked to an increased risk of port site infections following laparoscopic cholecystectomy. Hypertension, diabetes, hypothyroidism, acute cholecystitis, and spillage of bile during the procedure were all strongly associated with a higher risk of infection (12,13) as our first patient has diabetes and is currently receiving treatment he is at increased risk for port site infection. However, gender, age, and obesity were not found to be associated with an increased risk (14,15).

While all port sites can be affected, the epigastric port is the most commonly involved site for infection. This is likely due to its frequent use during the laparoscopic procedure (5,8). However, other studies have reported a higher incidence of infection at the umbilical port, potentially attributed to contamination from umbilical and gallbladder flora (16).

While the timing of port site infections can vary, most of our patients presented with these infections within one month of their laparoscopic surgery, aligning with the common observation of early onset in many studies (5,17).

The causative agent for port site infections can vary. However, bacteria are responsible for the majority of these infections, accounting for 55.17 % of cases. Port site tuberculosis represents a smaller proportion of infections, accounting for 5.17 % of port site infections and 0.2 % of all 1478 laparoscopic patients in the study (8).

Port site tuberculosis can be transmitted to patients through two main routes: exogenous, where improper sterilization of laparoscopic instruments introduces the bacteria, or endogenous, where it originates from a distant site within the patient's body and spreads to the surgical site (18,19). Given that our patients developed port site TB at the same center during the same campaign, the exogenous route, likely involving improper sterilization of laparoscopic instruments, is the more probable mode of transmission.

The most common presentation of port site TB as in our patients was wound discharge, as observed in the 55-year-old female patient (20), 65-year-old woman (21) and 28-year-old female patient (9); who was diagnosed with port site TB following a laparoscopic cholecystectomy.

The diagnosis of port site tuberculosis can be confirmed through various methods, including positive results for acid-fast bacilli (AFB), gene Xpert testing, culture, or histopathological examination (22,23). In our first two patients, FNAC revealed granulomatous inflammation, while the third patient had a positive AFB stain, supporting the diagnosis of port site TB. Microbiological confirmation of tuberculosis using culture and GeneXpert was not possible in this study due to blood contamination of the samples (for gene expert) and limitations in our laboratory setup (for culture).

Once a diagnosis of port site tuberculosis is confirmed through supportive history, exclusion of other bacterial infections, and confirmatory diagnostic tests, the next step involves treatment with a standard anti-tuberculosis regimen, typically consisting of 4 months RHZE followed by 2 months RH, along with nutritional rehabilitation (8). All of our patients responded favorably to this treatment approach.

## 4 Conclusion

Given that all our patients developed port site TB after undergoing laparoscopic cholecystectomy at the same facility, it's highly likely that inadequate instrument sterilization played a major role in their infections but other reasons may also play a role like reactivation of intra abdomina TB reactivation following surgery or hospital acquired infection due to poor infection control. This case underscores the importance of considering port site TB as a differential diagnosis in post-laparoscopic patients who do not respond to antibiotics, especially in low socioeconomic settings like Ethiopia, where TB is more prevalent. After carefully excluding bacterial infections, investigating for port site TB becomes crucial. Appropriate anti-TB management has shown excellent outcomes for patients, highlighting the importance of strict infection prevention protocols.

## Abbreviations

AFB: Acid-fast bacillus
HCT: hematocrit
Hgb: hemoglobin
PLT: Platelet
TB: Tuberculosis
2RHZE: Isoniazid, Rifampin, Ethambutol, and Pyrazinamide for 2 months
4RH: Rifampicin and Isoniazid for 4 months
WBC: white blood cell

## Consent

Written informed consent was obtained from the patient for publication and any accompanying images. A copy of the written consent is available for review by the Editor-in-Chief of this journal on request.

## Ethical approval

Ethical approval for this study was provided by the Department of Surgery Ethical review committee (SUR 19/2024), Debre Tabor University, Ethiopia, on September 23,2024.

## Research registration number

N/A

## Declaration of Generative AI and AI-assisted technologies in the writing process

AI language modelling tools were utilized for the improvement of English-language only in this case series.

## Source of funding

There is no source of funding found for this research paper.

## Author contribution

AAA: Conceptualization, design of the study, acquisition of data, drafting the article, revising it critically for important intellectual content, approval of the version to be submitted.

MW: Analysis, interpretation of data, drafting the article, revising it critically for important intellectual content, approval of the version to be submitted.

ZA: Conceptualization, analysis, drafting the article, revising it critically for important intellectual content, approval of the version to be submitted.

YG: Acquisition of data, analysis, revising it critically for important intellectual content, approval of the version to be submitted.

AAY: Acquisition of data, analysis, revising it critically for important intellectual content, approval of the version to be submitted.

AMG: Acquisition of data, analysis, revising it critically for important intellectual content, approval of the version to be submitted.

## Guarantors

Mulugeta Wondmu Kedimu,MD

Addisu Assfaw Ayen, MD

## Declaration of competing interest

All authors declare that they have no conflict of interest.
